# Supplementary material for: Optimized nickase- and nuclease-based prime editing in human and mouse cells
Source: Nucleic Acids Res. 2021 Sep 17;49(18):10785–95. doi: 10.1093/nar/gkab792 (PMC8501948; doi:10.1093/nar/gkab792)
Supplement: gkab792_Supplemental_File [file gkab792_supplemental_file.pdf]

Supplementary Data.

Optimized nickase- and nuclease-based prime editing in human and mouse cells

Fatwa Adikusuma[1,2,5,†,*], Caleb Lushington[1,2,†], Jayshen Arudkumar[1,2,†], Gelshan I. Godahewa[2,6,†], Yu C.J. Chey[1,2,†], Luke Gierus[1,2], Sandra Piltz[1,2,3], Ashleigh Geiger[1,2,5], Yatish Jain[4,8], Daniel Reti[4,8], Laurence OW. Wilson[4,8], Denis C. Bauer[4,7,8] and Paul Q. Thomas[1,2,3,*]

[1]School of Biomedicine and Robinson Research Institute, University of Adelaide, Adelaide, SA, Australia

[2]Genome Editing Program, South Australian Health and Medical Research Institute (SAHMRI), Adelaide, SA, Australia

[3]South Australian Genome Editing (SAGE), South Australian Health and Medical Research Institute (SAHMRI), Adelaide, SA, Australia

[4]Australian e-Health Research Centre, CSIRO, North Ryde, Australia

[5]CSIRO Synthetic Biology Future Science Platform, Australia

[6]Australian Centre for Disease Preparedness, CSIRO Health and Biosecurity, Geelong, VIC, Australia

[7]Department of Biomedical Sciences, Macquarie University, New South Wales, Sydney, Australia

[8]Applied BioSciences, Faculty of Science and Engineering, Macquarie University, New South Wales, Sydney, Australia

[*] To whom correspondence should be addressed
paul.thomas@adelaide.edu.au (P.Q.T)
Correspondence may also be addressed to
fatwa.adikusuma@adelaide.edu.au (F.A)

[†]Joint first authors

Supplementary Figure S1. Sequence view of hU6-pegRNA and hU6-gRNA (second-nick) cassettes and the golden gate cloning sites in PEA1 construct.

Benchling sequence view of hU6-pegRNA cassette of PEA1

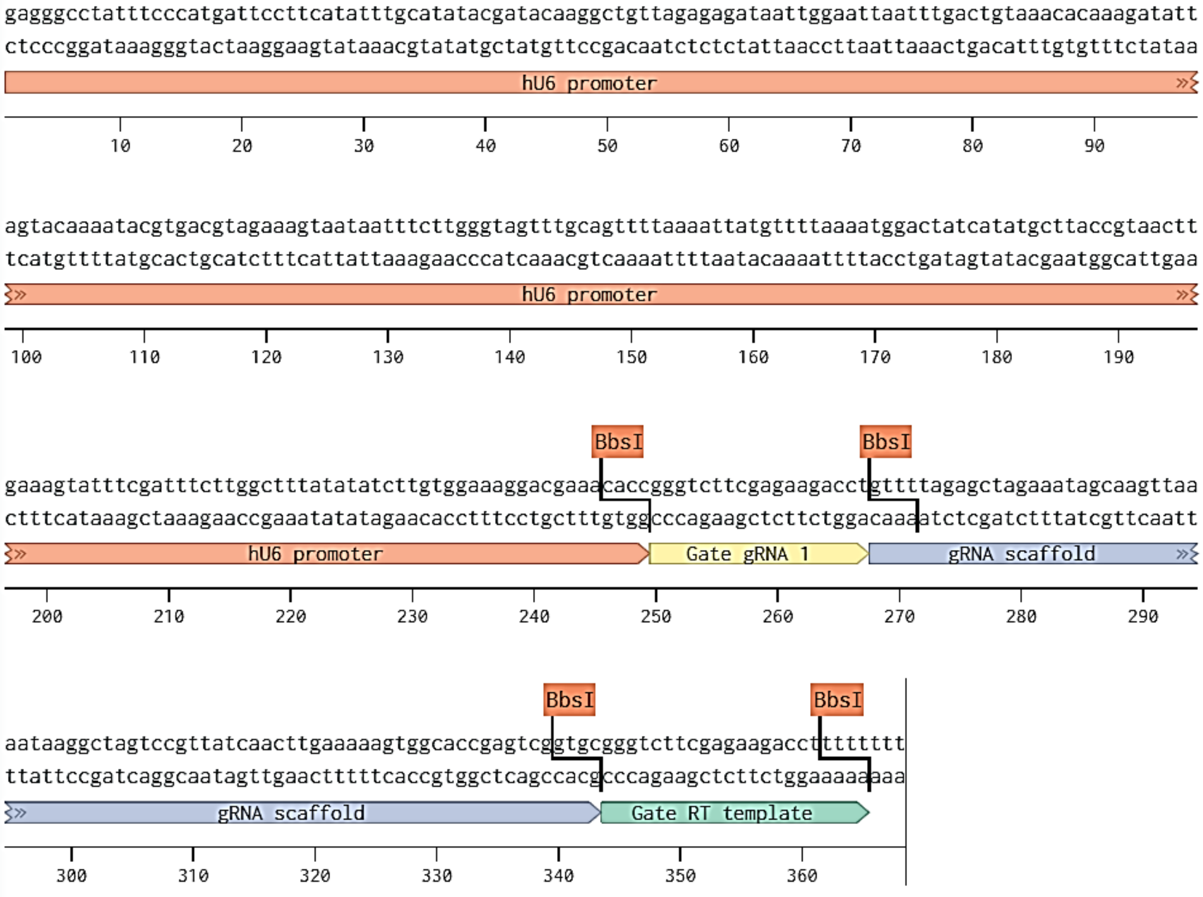

Benchling sequence view of hU6-gRNA (second-nick) cassette of PEA1

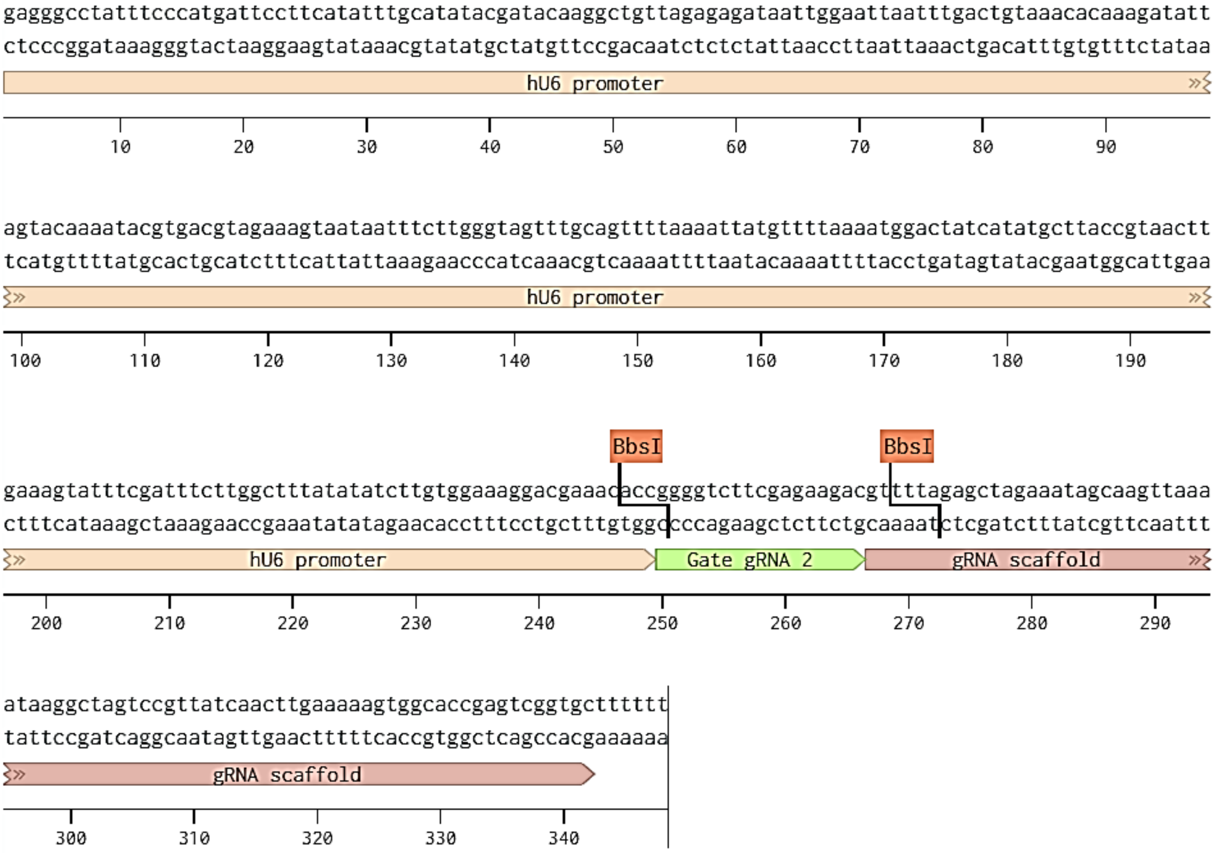

Supplementary Figure S2. Example of Bbs1 check digest of plasmids resulting from one-step digestion-ligation cloning to generate PEA1-Puro *VEGFA* +4 C ins. All plasmids except for plasmid 2 and plasmid 6 had complete integration of the oligo pairs. Plasmid 6 seemed to lack one pair of oligo duplex integration and therefore could be digested and produced linear plasmid.

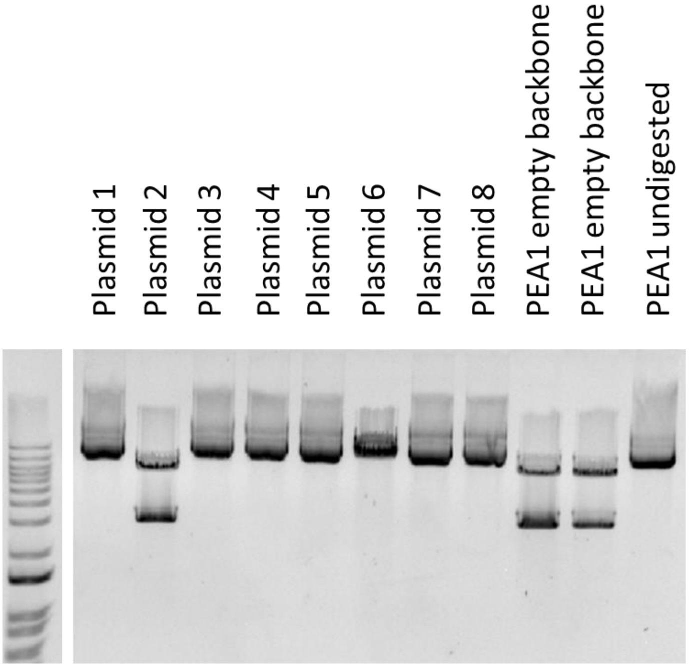

Supplementary Figure S3. Prime Editing Target Locator (PETAL) – an online tool for designing prime editing applications. As input, PETAL takes the target sequence to be edited (top) as well as the final edited sequence (bottom, new inserted sequence highlighted in blue). PETAL identifies all valid guide sequences within the target sequence (top, edited sequence highlighted in dark grey). Users are then able to select the desired pegRNA (blue) and Second Nick (yellow) sequences. PETAL then provides the necessary oligo sequences for both gRNA's as well as those required for the template strand (bottom). The density plot enables users to zoom into the regions of interest.

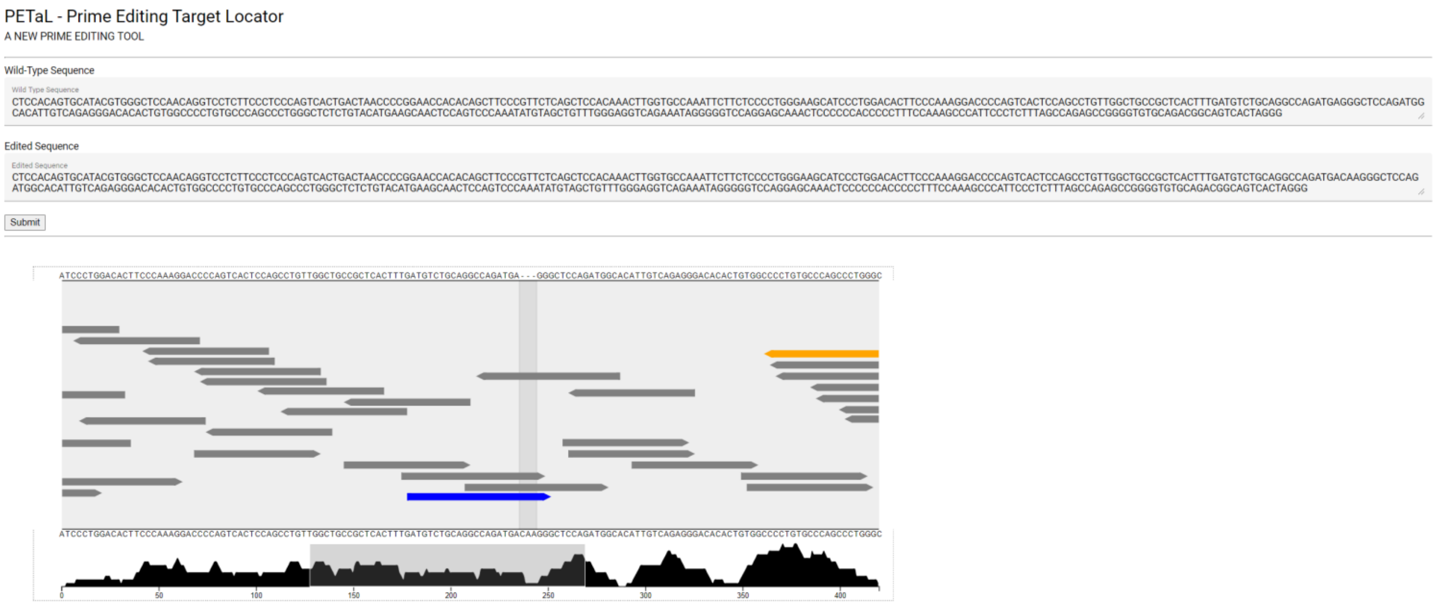

Target Selection and Oligos:

Guide Type	Name	Sequence
pegRNA	Guide	GATGTCTGCAGGCCAGATGA
pegRNA	Oligo #1	caccGATGTCTGCAGGCCAGATGA
pegRNA	Oligo #2	aaacTCATCTGGCCTGCAGACATC
Second Nick	Guide	gAGAGCCCAGGGCTGGGCACA
Second Nick	Oligo #1	taaaacTGTGCCCAGCCCTGGGCTCT
Second Nick	Oligo #2	accgAGAGCCCAGGGCTGGGCACAgt
Template	Guide	CATCTGGAGCCCTTGTCATCTGGCCTGC
Template	Oligo #1	gtgcCATCTGGAGCCCTTGTCATCTGGCCTGC
Template	Oligo #2	aaaaGCAGGCCAGATGACAAGGGCTCCAGATG

Supplementary Figure S4. Mechanism of partial template duplication (PTD) events.

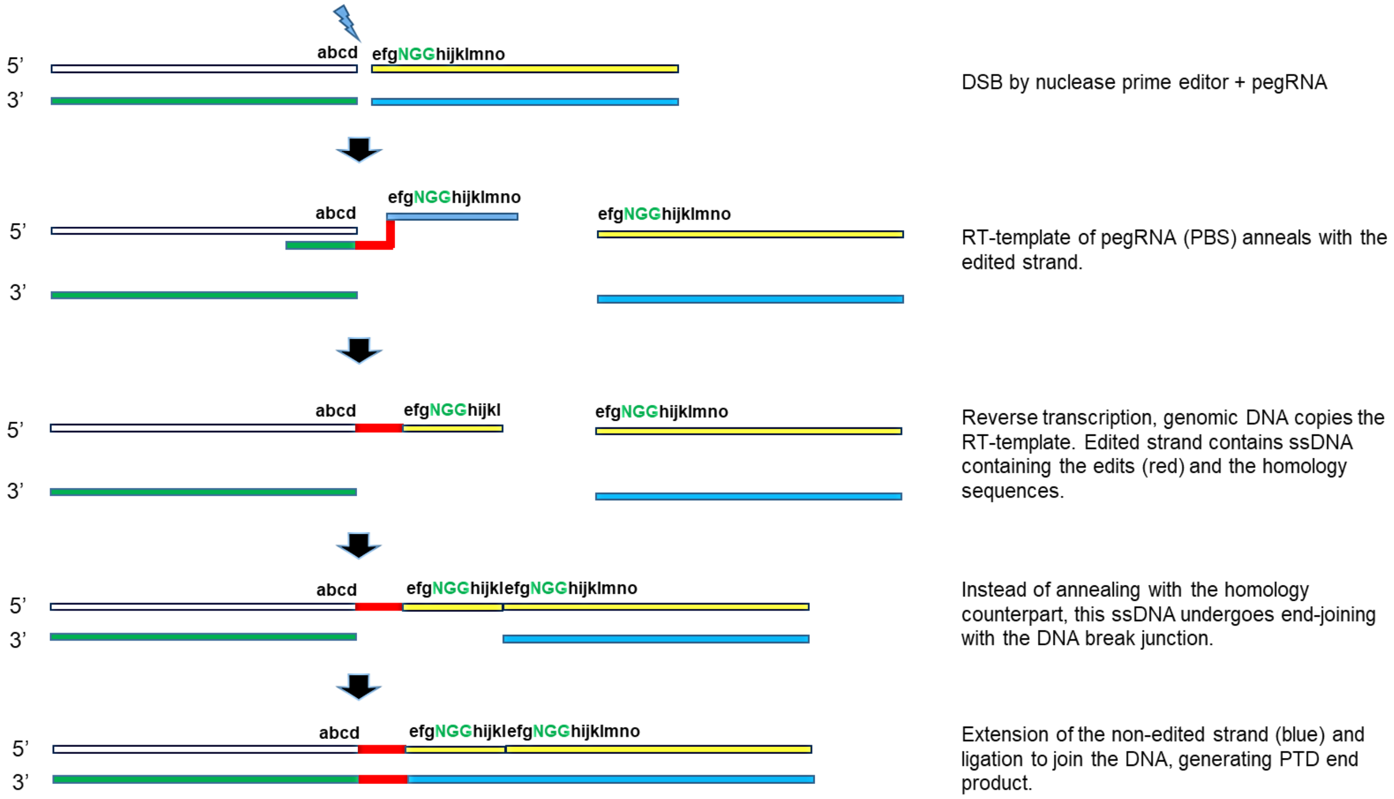

Supplementary Figure S5. Partial template duplications were also observed as unintended editing outcomes of PE3 in HEK293T cells.

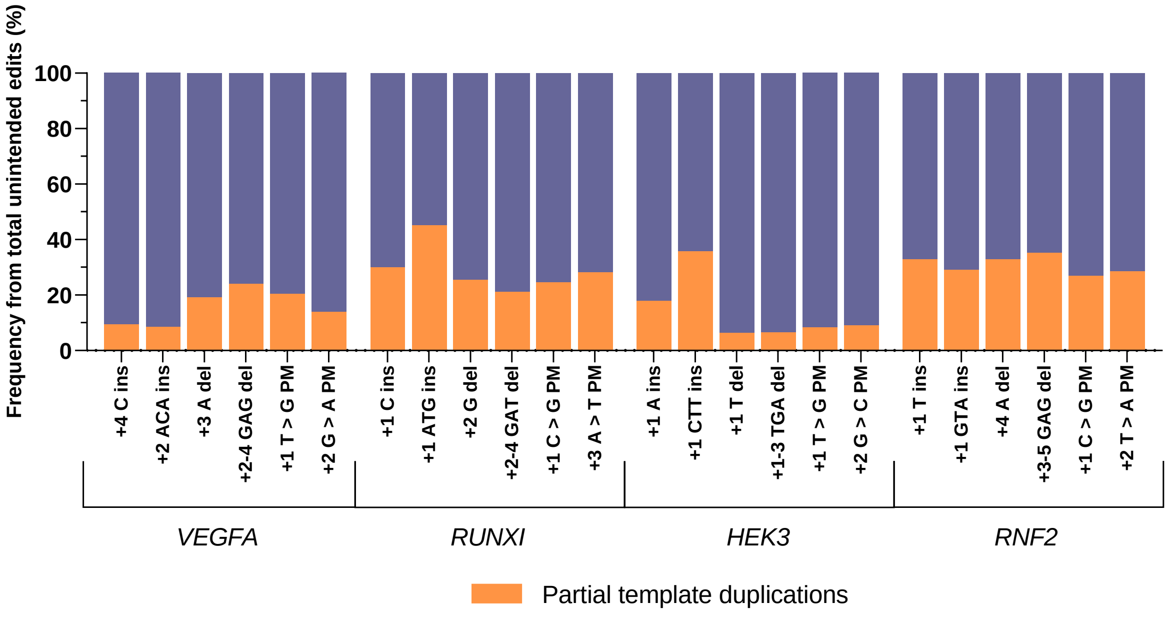

Supplementary Figure S6. Examples of edits found in mouse ES cells targeted with PEA1-Nuc Col12a1 +2 A to C that indicate re-cutting events after prime editing events.

```
TGCCCTGATTAAACCTATTGGAAGAGCTTGACTTCCATGGTTCCACAATGGGTCCATTATGTGCTGGGCTGGGCCCTGTTGTTTTATA
||||||||||||||||||||||||||||||||||           |.||||||||||||||||||||||||||||||||||||||||
TGCCCTGATTAAACCTATTGGAAGAGCTTGACTT-----------CCATGGGTCCATTATGTGCTGGGCTGGGCCCTGTTGTTTTATA
```

14.37%
(n=3)

```
TGCCCTGATTAAACCTATTGGAAGAGCTTGACTTCCATGGTTCCACAATGGGTCCATTATGTGCTGGGCTGGGCCCTGTTGTTTTATA
||||||||||||||||||||||||||||||||||||||||||   |.||||||||||||||||||||||||||||||||||||||||
TGCCCTGATTAAACCTATTGGAAGAGCTTGACTTCCATGGTT---CCATGGGTCCATTATGTGCTGGGCTGGGCCCTGTTGTTTTATC
```

2.77%
(n=3)

Supplementary Figure S7. Frequency of PE, indels, PTD and WT alleles in individual mice generated by nuclease prime editor.

# indicates 3 or more different alleles were detected in this individual mouse.
Alleles with 2 or more bp non-correct insertions were classified as PTDs.

PE-Nuc mice *Chd2* +1 CTC ins

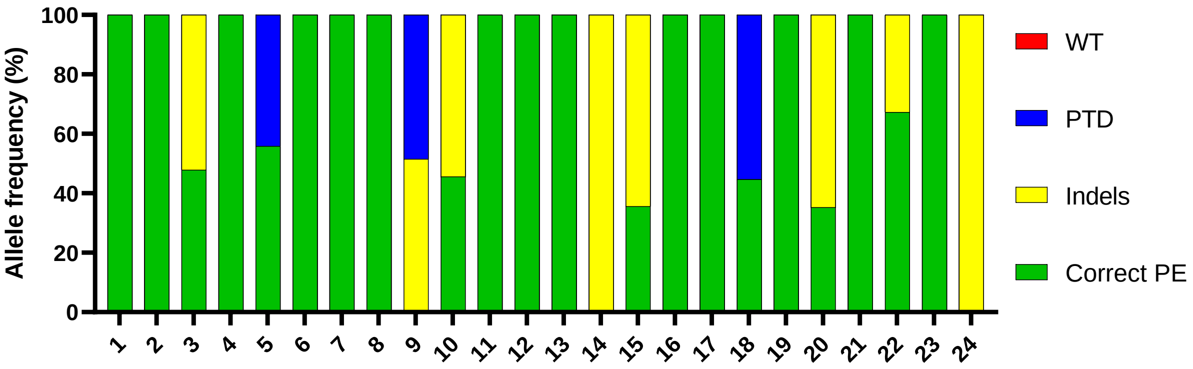

PE-Nuc mice *Chd2* +5 G to C

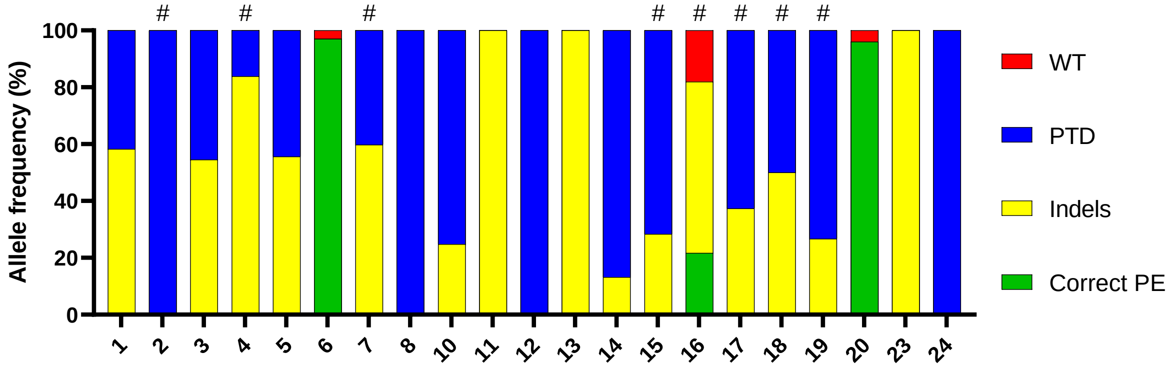

PE-Nuc mice *Col12a1* +1 GTG ins

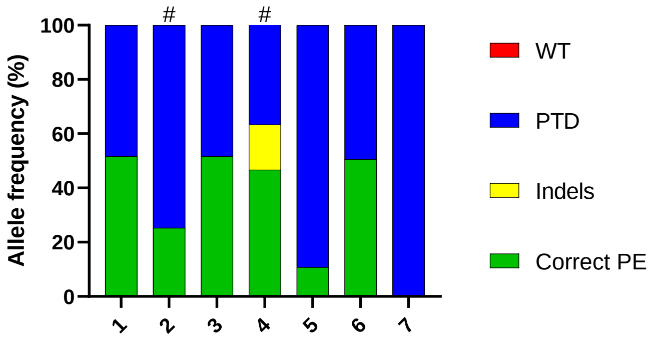

PE-Nuc mice *Col12a1* +2 A to C

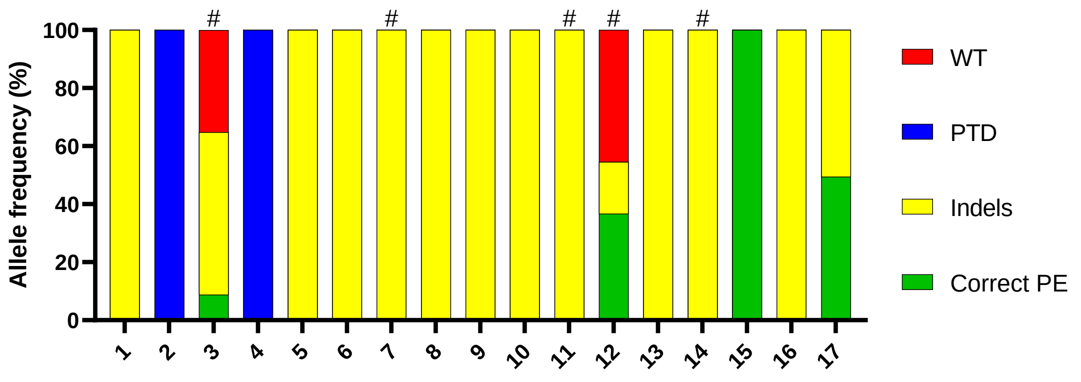

PE-Nuc mice *Col12a1* +1-3 CAA to ACC

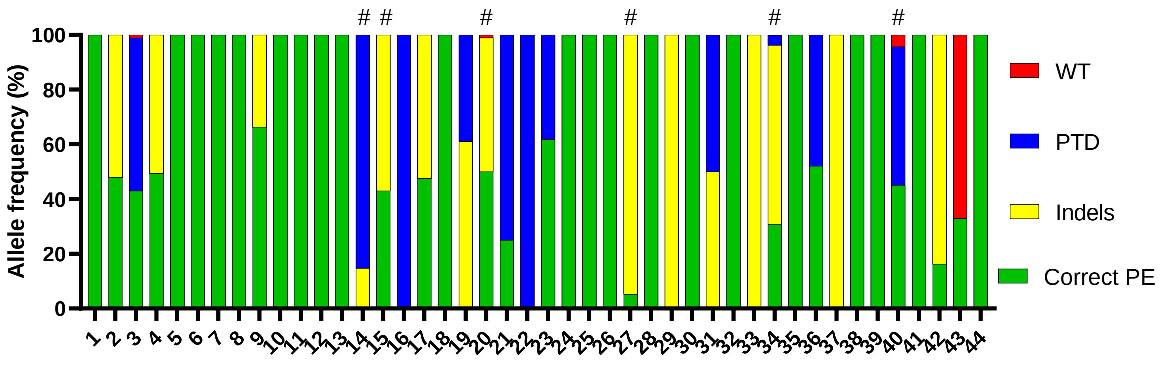

PE-Nuc mice *Tyr* +1 TGT ins

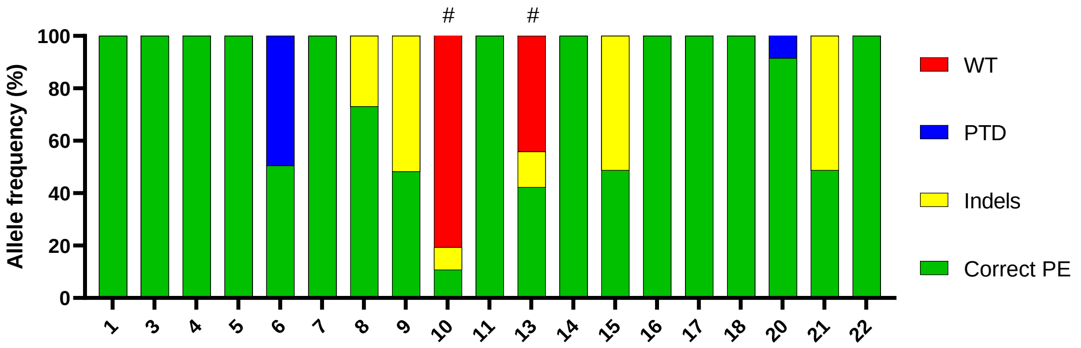

PE-Nuc mice *Tyr* +6 G to A

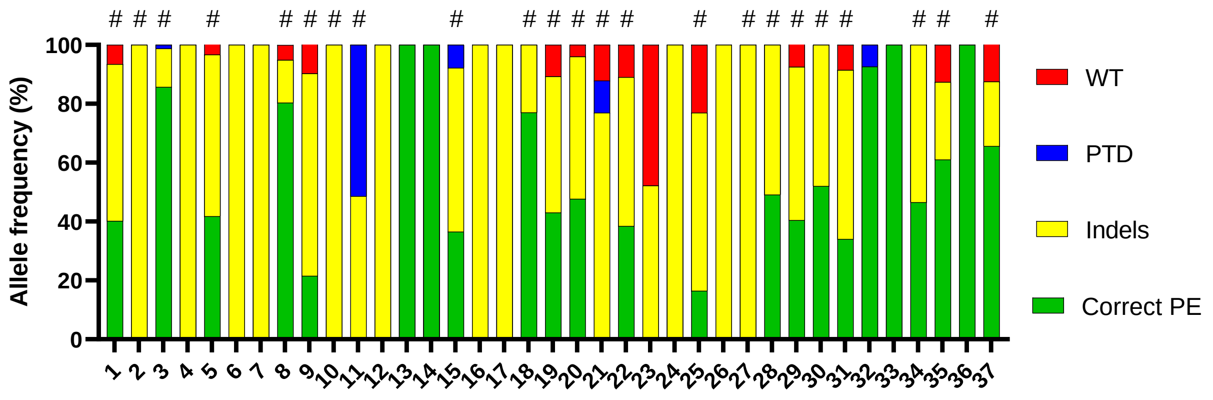

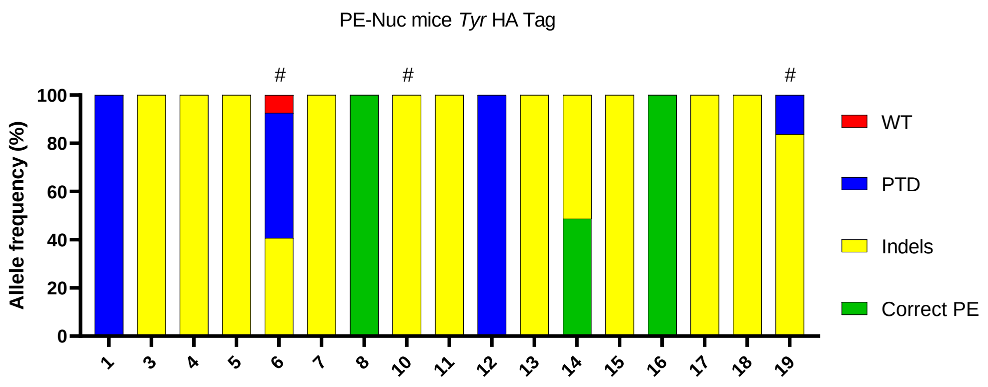
PE-Nuc mice *Tyr* HA Tag
Allele frequency (%)
0
20
40
60
80
100
#
#
#
1
3
4
5
6
7
8
10
11
12
13
14
15
16
17
18
19
WT
PTD
Indels
Correct PE

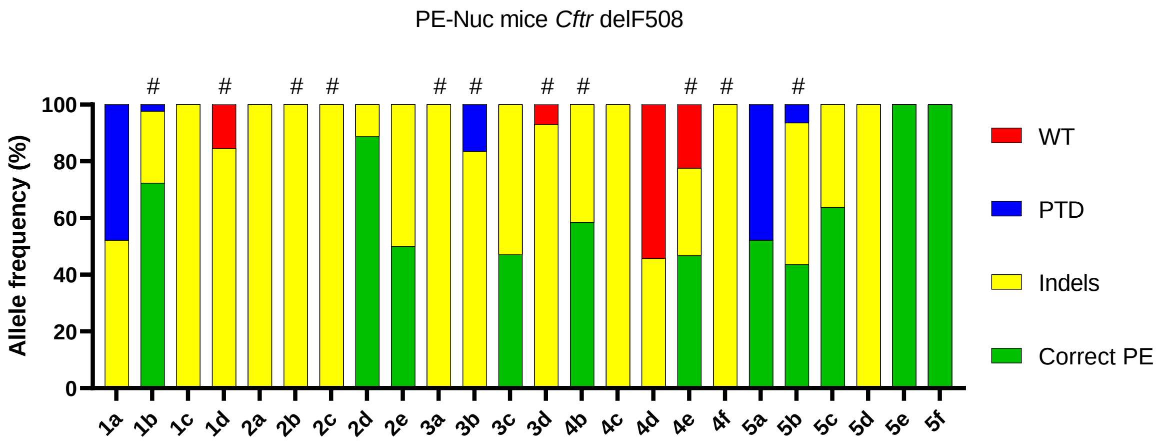
PE-Nuc mice *Cftr* delF508
Allele frequency (%)
0
20
40
60
80
100
#
#
#
#
#
#
#
#
#
#
#
1a
1b
1c
1d
2a
2b
2c
2d
2e
3a
3b
3c
3d
4b
4c
4d
4e
4f
5a
5b
5c
5d
5e
5f
WT
PTD
Indels
Correct PE

Supplementary Table S1. Oligo lists to generate the PEA1 targeting constructs.

PEA1 targeting construct	Oligo pair 1 (gRNA)	Oligo pair 2 (repair template)	Oligo pair 3 (second-nick gRNA)
HEK3 +1 A ins	caccGGCCCAGACTGAGCACGTGA	gtgcTCTGCCATCATCGTGCTCAGTCTG	accgTCAACCAGTATCCCGGTGCgt
	aaacTCACGTGCTCAGTCTGGGCC	aaaaCAGACTGAGCACGATGATGGCAGA	taaaacGCACCGGGATACTGGTTGA
HEK3 +1 CTT ins	caccGGCCCAGACTGAGCACGTGA	gtgcTCTGCCATCAAAGCGTGCTCAGTCTG	accgTCAACCAGTATCCCGGTGCgt
	aaacTCACGTGCTCAGTCTGGGCC	aaaaCAGACTGAGCACGCTTTGATGGCAGA	taaaacGCACCGGGATACTGGTTGA
HEK3 +1 T del	caccGGCCCAGACTGAGCACGTGA	gtgcTCTGCCATCCGTGCTCAGTCTG	accgTCAACCAGTATCCCGGTGCgt
	aaacTCACGTGCTCAGTCTGGGCC	aaaaCAGACTGAGCACGGATGGCAGA	taaaacGCACCGGGATACTGGTTGA
HEK3 +1-3 TGA del	caccGGCCCAGACTGAGCACGTGA	gtgcTGGAGGAAGCAGGGCTTCCTTTCCTCTGCCACGTGCTCAGTCTG	accgTCAACCAGTATCCCGGTGCgt
	aaacTCACGTGCTCAGTCTGGGCC	aaaaCAGACTGAGCACGTGGCAGAGGAAAGGAAGCCCTGCTTCCTCCA	taaaacGCACCGGGATACTGGTTGA
HEK3 +1 T to G PM	caccGGCCCAGACTGAGCACGTGA	gtgcTCTGCCATCCCGTGCTCAGTCTG	accgTCAACCAGTATCCCGGTGCgt
	aaacTCACGTGCTCAGTCTGGGCC	aaaaCAGACTGAGCACGGGATGGCAGA	taaaacGCACCGGGATACTGGTTGA
HEK3 +2 G to C	caccGGCCCAGACTGAGCACGTGA	gtgcTCTGCCATGACGTGCTCAGTCTG	accgTCAACCAGTATCCCGGTGCgt
	aaacTCACGTGCTCAGTCTGGGCC	aaaaCAGACTGAGCACGTCATGGCAGA	taaaacGCACCGGGATACTGGTTGA
RNF2 +1 T ins	caccGTCATCTTAGTCATTACCTG	gtgcAACGAACACCTCAGAGTAATGACTAAGATG	accgTCAACCATTAAGCAAAACATgt
	aaacCAGGTAATGACTAAGATGAC	aaaaCATCTTAGTCATTACTCTGAGGTGTTCGTT	taaaacATGTTTTGCTTAATGGTTGA
RNF2 +1 GTA ins	caccGTCATCTTAGTCATTACCTG	gtgcAACGAACACCTCAGTACGTAATGACTAAGATG	accgTCAACCATTAAGCAAAACATgt
	aaacCAGGTAATGACTAAGATGAC	aaaaCATCTTAGTCATTACGTACTGAGGTGTTCGTT	taaaacATGTTTTGCTTAATGGTTGA
RNF2 +4 A del	caccGTCATCTTAGTCATTACCTG	gtgcAACGAACACCCAGGTAATGACTAAGATG	accgTCAACCATTAAGCAAAACATgt
	aaacCAGGTAATGACTAAGATGAC	aaaaCATCTTAGTCATTACCTGGGTGTTCGTT	taaaacATGTTTTGCTTAATGGTTGA
RNF2 +3-5 GAG del	caccGTCATCTTAGTCATTACCTG	gtgcAACGAACACAGGTAATGACTAAGATG	accgTCAACCATTAAGCAAAACATgt
	aaacCAGGTAATGACTAAGATGAC	aaaaCATCTTAGTCATTACCTGTGTTCGTT	taaaacATGTTTTGCTTAATGGTTGA
RNF2 +1 C to G	caccGTCATCTTAGTCATTACCTG	gtgcAACGAACACCTCACGTAATGACTAAGATG	accgTCAACCATTAAGCAAAACATgt
	aaacCAGGTAATGACTAAGATGAC	aaaaCATCTTAGTCATTACGTGAGGTGTTCGTT	taaaacATGTTTTGCTTAATGGTTGA
RNF2 +2 T to A	caccGTCATCTTAGTCATTACCTG	gtgcAACGAACACCTCTGGTAATGACTAAGATG	accgTCAACCATTAAGCAAAACATgt
	aaacCAGGTAATGACTAAGATGAC	aaaaCATCTTAGTCATTACCAGAGGTGTTCGTT	taaaacATGTTTTGCTTAATGGTTGA
RUNX1 +1 C ins	caccGCATTTTCAGGAGGAAGCGA	gtgcTGTCTGAAGCCATCGGCTTCCTCCTGAAAAT	accgATGAAGCACTGTGGGTACGAgt
	aaacTCGCTTCCTCCTGAAAATGC	aaaaATTTTCAGGAGGAAGCCGATGGCTTCAGACA	taaaacTCGTACCCACAGTGCTTCAT
RUNX1 +1 ATG ins	caccGCATTTTCAGGAGGAAGCGA	gtgcTGTCTGAAGCCATCCATGCTTCCTCCTGAAAAT	accgATGAAGCACTGTGGGTACGAgt
	aaacTCGCTTCCTCCTGAAAATGC	aaaaATTTTCAGGAGGAAGCATGGATGGCTTCAGACA	taaaacTCGTACCCACAGTGCTTCAT
RUNX1 +2 G del	caccGCATTTTCAGGAGGAAGCGA	gtgcTGTCTGAAGCCATGCTTCCTCCTGAAAAT	accgATGAAGCACTGTGGGTACGAgt
	aaacTCGCTTCCTCCTGAAAATGC	aaaaATTTTCAGGAGGAAGCATGGCTTCAGACA	taaaacTCGTACCCACAGTGCTTCAT
RUNX1 +2-4 GAT del	caccGCATTTTCAGGAGGAAGCGA	gtgcTGTCTGAAGCCGCTTCCTCCTGAAAAT	accgATGAAGCACTGTGGGTACGAgt
	aaacTCGCTTCCTCCTGAAAATGC	aaaaATTTTCAGGAGGAAGCGGCTTCAGACA	taaaacTCGTACCCACAGTGCTTCAT
RUNX1 +1 C to G	caccGCATTTTCAGGAGGAAGCGA	gtgcTGTCTGAAGCCATCCCTTCCTCCTGAAAAT	accgATGAAGCACTGTGGGTACGAgt
	aaacTCGCTTCCTCCTGAAAATGC	aaaaATTTTCAGGAGGAAGGGATGGCTTCAGACA	taaaacTCGTACCCACAGTGCTTCAT

RUNX1 +3 A to T	caccGCATTTTCAGGAGGAAGCGA	gtgcTGTCTGAAGCCAACGCTTCCTCCTGAAAAT	accgATGAAGCACTGTGGGTACGAgt
	aaacTCGCTTCCTCCTGAAAATGC	aaaaATTTTCAGGAGGAAGCGTTGGCTTCAGACA	taaaacTCGTACCCACAGTGCTTCAT
VEGFA +4 C ins	caccGATGTCTGCAGGCCAGATGA	gtgcAATGTGCCATCTGGAGCCGCTCATCTGGCCTGCAGA	accgATGTACAGAGAGCCCAGGGCgt
	aaacTCATCTGGCCTGCAGACATC	aaaaTCTGCAGGCCAGATGAGCGGCTCCAGATGGCACATT	taaaacGCCCTGGGCTCTCTGTACAT
VEGFA +2 ACA ins	caccGATGTCTGCAGGCCAGATGA	gtgcAATGTGCCATCTGGAGCCCTTGTCATCTGGCCTGCAGA	accgATGTACAGAGAGCCCAGGGCgt
	aaacTCATCTGGCCTGCAGACATC	aaaaTCTGCAGGCCAGATGACAAGGGCTCCAGATGGCACATT	taaaacGCCCTGGGCTCTCTGTACAT
VEGFA +3 A del	caccGATGTCTGCAGGCCAGATGA	gtgcAATGTGCCATCTGGAGCCCCATCTGGCCTGCAGA	accgATGTACAGAGAGCCCAGGGCgt
	aaacTCATCTGGCCTGCAGACATC	aaaaTCTGCAGGCCAGATGGGGCTCCAGATGGCACATT	taaaacGCCCTGGGCTCTCTGTACAT
VEGFA +2-4 GAG del	caccGATGTCTGCAGGCCAGATGA	gtgcAATGTGCCATCTGGAGCCATCTGGCCTGCAGA	accgATGTACAGAGAGCCCAGGGCgt
	aaacTCATCTGGCCTGCAGACATC	aaaaTCTGCAGGCCAGATGGCTCCAGATGGCACATT	taaaacGCCCTGGGCTCTCTGTACAT
VEGFA +1 T to G	caccGATGTCTGCAGGCCAGATGA	gtgcAATGTGCCATCTGGAGCCCTCCTCTGGCCTGCAGA	accgATGTACAGAGAGCCCAGGGCgt
	aaacTCATCTGGCCTGCAGACATC	aaaaTCTGCAGGCCAGAGGAGGGCTCCAGATGGCACATT	taaaacGCCCTGGGCTCTCTGTACAT
VEGFA +2 G to A	caccGATGTCTGCAGGCCAGATGA	gtgcAATGTGCCATCTGGAGCCCTTATCTGGCCTGCAGA	accgATGTACAGAGAGCCCAGGGCgt
	aaacTCATCTGGCCTGCAGACATC	aaaaTCTGCAGGCCAGATAAGGGCTCCAGATGGCACATT	taaaacGCCCTGGGCTCTCTGTACAT
Chd2 +1 CTC ins	caccGCGGTAGCTCCCAGAACGGT	gtgcGATGCCCACCgagGTTCTGGGAGCTA	accgACCATCAGTATGAGCAGCATgt
	aaacACCGTTCTGGGAGCTACCGC	aaaaTAGCTCCCAGAACctcGGTGGGCATC	taaaacATGCTGCTCATACTGATGGT
Chd2 +5 G to C	caccGCGGTAGCTCCCAGAACGGT	gtgcGATGCgCACCGTTCTGGGAGCTA	accgACCATCAGTATGAGCAGCATgt
	aaacACCGTTCTGGGAGCTACCGC	aaaaTAGCTCCCAGAACGGTGcGCATC	taaaacATGCTGCTCATACTGATGGT
Col12a1 +1 GTG ins	caccGACTTCCATGGTTCCACAA	gtgcAATGGACCCATTGcacTGGAACCATGGAA	accgCCTGAGCAGGCCACGAACAgt
	aaacTTGTGGAACCATGGAAGTC	aaaaTTCCATGGTTCCAgtgCAATGGGTCCATT	taaaacTGTTCGTGGCCTGCTCAGG
Col12a1 +2 A to C	caccGACTTCCATGGTTCCACAA	gtgcAATGGACCCATgGTGGAACCATGGAA	accgCCTGAGCAGGCCACGAACAgt
	aaacTTGTGGAACCATGGAAGTC	aaaaTTCCATGGTTCCACcATGGGTCCATT	taaaacTGTTCGTGGCCTGCTCAGG
Col12a1 +1-3 CAA to ACC	caccGACTTCCATGGTTCCACAA	gtgcAATGGACCCAggtTGGAACCATGGAA	accgGGCAGCGCGGCTATCGTGGCgt
	aaacTTGTGGAACCATGGAAGTC	aaaaTTCCATGGTTCCAaccTGGGTCCATT	taaaacGCCACGATAGCCGCGCTGCC
Tyr +1 TGT ins	caccGCAAAAGAATGCTGCCCACCA	gtgcATCACCCATCCATGGacaTGGGCAGCATTCT	accgCACTGGACAGAAGGATATCCgt
	aaacTGGTGGGCAGCATTCTTTTGC	aaaaAGAATGCTGCCCAtgtCCATGGATGGGTGAT	taaaacGGATATCCTTCTGTCCAGTG
Tyr +6 G to A	caccGCAAAAGAATGCTGCCCACCA	gtgcATCACCCATtCATGGTGGGCAGCATTCT	accgCACTGGACAGAAGGATATCCgt
	aaacTGGTGGGCAGCATTCTTTTGC	aaaaAGAATGCTGCCCACCATGaATGGGTGAT	taaaacGGATATCCTTCTGTCCAGTG
Tyr HA-Tag	caccGTTTCCTAGGATGTTCACAGA	gtgcTCAGAGCCATCTgTACCCATACGATGTTCCAGATTACGCTtaaGTGAACATCCTAG	accgGGCAGCGCGGCTATCGTGGCgt
	aaacTCTGTGAACATCCTAGGAAAC	aaaaCTAGGATGTTCACttaAGCGTAATCTGGAACATCGTATGGGTAcAGATGGCTCTGA	taaaacGCCACGATAGCCGCGCTGCC
Mixl1 +1 CTT ins (Nick +48)	caccGCAAGTGGATGTCTGGGTACA	gtgcTCCGACAGACCATGTaagACCCAGACATCCAC	accgCAAGCGCACGTCGTTCAGCTgt
	aaacTGTACCCAGACATCCACTTGC	aaaaGTGGATGTCTGGGTcttACATGGTCTGTCGGA	taaaacAGCTGAACGACGTGCGCTTG
Mixl1 +1 A to G (Nick +48)	caccGCAAGTGGATGTCTGGGTACA	gtgcTCCGACAGACCATGcACCCAGACATCCAC	accgCAAGCGCACGTCGTTCAGCTgt
	aaacTGTACCCAGACATCCACTTGC	aaaaGTGGATGTCTGGGTgCATGGTCTGTCGGA	taaaacAGCTGAACGACGTGCGCTTG
Mixl1 +1-3 ACA del (Nick +48)	caccGCAAGTGGATGTCTGGGTACA	gtgcTCCGACAGACCAACCCAGACATCCAC	accgCAAGCGCACGTCGTTCAGCTgt
	aaacTGTACCCAGACATCCACTTGC	aaaaGTGGATGTCTGGGTTGGTCTGTCGGA	taaaacAGCTGAACGACGTGCGCTTG

Mixl1<br>+1 CTT ins<br>(Nick -60)	caccGCAAGTGGATGTCTGGGTACA	gtgcTCCGACAGACCATGTaagACCCAGACATCCAC	accgCTACCCGAGTCCAGGATCCgt
	aaacTGTACCCAGACATCCACTTGC	aaaaGTGGATGTCTGGGTcttACATGGTCTGTCGGA	taaaacGGATCCTGGACTCGGGTAG
Mixl1<br>+1 A to G (Nick -60)	caccGCAAGTGGATGTCTGGGTACA	gtgcTCCGACAGACCATGcACCCAGACATCCAC	accgCTACCCGAGTCCAGGATCCgt
	aaacTGTACCCAGACATCCACTTGC	aaaaGTGGATGTCTGGGTgCATGGTCTGTCGGA	taaaacGGATCCTGGACTCGGGTAG
Mixl1<br>+1-3 ACA del<br>(Nick -60)	caccGCAAGTGGATGTCTGGGTACA	gtgcTCCGACAGACCAACCCAGACATCCAC	accgCTACCCGAGTCCAGGATCCgt
	aaacTGTACCCAGACATCCACTTGC	aaaaGTGGATGTCTGGGTTGGTCTGTCGGA	taaaacGGATCCTGGACTCGGGTAG
EphB2<br>loxP site 1 (R)	caccGCCATGGTCTCAGGTAATAGC	gtgcTTGTCTCAGCTCCTGCTATAACTTCGTATAATGTATGCTATACGAAGTTATcaattgATTACCTGAGACCA	accgAGAGAAAGATGAGACTGGAgt
	aaacGCTATTACCTGAGACCATGGC	aaaaTGGTCTCAGGTAATcaattgATAACTTCGTATAGCATACATTATACGAAGTTATAGCAGGAGCTGAGACAA	taaaacTCCAGTCTCATCTTTCTCT
EphB2<br>loxP site 2 (L2)	caccGCAGTCACTCTGTAAACCCTG	gtgcGAAGAGCGCGACCCCAGATAACTTCGTATAATGTATGCTATACGAAGTTATgatatcGGTTTACAGAGTGA	accgAGTATGGAGCAGAGAGGCTgt
	aaacCAGGGTTTACAGAGTGACTGC	aaaaTCACTCTGTAAACCgatatcATAACTTCGTATAGCATACATTATACGAAGTTATCTGGGGTCGCGCTCTTC	taaaacAGCCTCTCTGCTCCATACT
EphB2<br>loxP site 3 (L3)	caccGCCAAGAGCCTAGGCAATCGT	gtgcAGAGGTAGACTCCCACGATAACTTCGTATAATGTATGCTATACGAAGTTATgatatcATTGCCTAGGCTCT	accgCCACTCCACCAGTAAAGAAAgt
	aaacACGATTGCCTAGGCTCTTGGC	aaaaAGAGCCTAGGCAATgatatcATAACTTCGTATAGCATACATTATACGAAGTTATCGTGGGAGTCTACCTCT	taaaacTTTCTTTACTGGTGGAGTGG
Cftr<br>+1-3 CTT del	caccATCAAAGAAAATATCATCTT	gtgcATCATAGGAAACACCAATGATATTTTCTTTG	accgGGCAGCGCGGCTATCGTGGCgt
	aaacAAGATGATATTTTCTTTGAT	aaaaCAAAGAAAATATCATTGGTGTTTCCTATGAT	taaaacGCCACGATAGCCGCGCTGCC

gRNA sequences are underlined. Red highlight indicates extra G was added to the gRNA sequences. Oligo pair 3 for PEA1-Nuc targeting constructs used a sham targeting oligos which are the same oligos highlighted in blue).

Supplementary Table S2. Primers to generate IVT template of pegRNAs for mouse zygote injections.

Target	Forward primer	Reverse primer
Chd2 +1 CTC ins	TTAATACGACTCACTATAGGCGGTAGCTCCCAGAACGGT	aaaaTAGCTCCCAGAACctcGGTGGGCATC
Chd2 +5 G to C	TTAATACGACTCACTATAGGCGGTAGCTCCCAGAACGGT	aaaaTAGCTCCCAGAACGGTGcGCATC
Col12a1 +1 GTG ins	TTAATACGACTCACTATAGTGACTTCCATGGTTCCACAA	aaaaTTCCATGGTTCCAgtgCAATGGGTCCATT
Col12a1 +2 A to C	TTAATACGACTCACTATAGTGACTTCCATGGTTCCACAA	aaaaTTCCATGGTTCCACcATGGGTCCATT
Col12a1 +1-3 CAA to ACC	TTAATACGACTCACTATAGTGACTTCCATGGTTCCACAA	aaaaTTCCATGGTTCCAaccTGGGTCCATT
Tyr +1 TGT ins	TTAATACGACTCACTATAGCAAAAGAATGCTGCCCACCA	aaaaAGAATGCTGCCCAtgtCCATGGATGGGTGAT
Tyr +6 G to A	TTAATACGACTCACTATAGCAAAAGAATGCTGCCCACCA	aaaaAGAATGCTGCCCACCATGaATGGGTGAT
Tyr HA-Tag	TTAATACGACTCACTATAGTTTCCTAGGATGTTCACAGA	aaaaCTAGGATGTTCACttaAGCGTAATCTGGAACATCGTATGGGTAcAGATGGCTCTGA
Cftr +1-3 CTT del	TTAATACGACTCACTATAGATCAAAGAAAATATCATCTT	aaaaCAAAGAAAATATCATTGGTGTTTCCTATGAT

T7 promoter sequences are highlighted in green. The reverse primers are the same as the bottom primers used for oligo pair 2 for generating PEA1 targeting constructs.

Supplementary Table S3. List of PCR primers for sequencing.

Target sites	Forward	Reverse
HEK3	GGGAAACGCCCATGCAATTA	CAGAGATCAACCAGATTACCCCA
RNF2	ACGTAGGAATTTTGGTGGGACA	ACAGATGTAGCACCAACCATGGA
RUNX1	AGAGAGATGTAGGGCTAGAGGG	CACTTGACAAAGTTCTCACGC
VEGFA	CTCCACAGTGCATACGTGGG	CCCTAGTGACTGCCGTCTG
Chd2	CTTGCAGATCGAGGAGACTGG	CTCTCCTGCATCCTCAGGCT
Col12a1	CAGTATGAAGTCATGTGCGGTC	CAATGGAAGACAGGAGTAGGGC
Tyr	GTCTGTGACACTCATTAACCTATTGGTGC	TCAACTGCGGAAACTGTAAGTTTGGA
Tyr-HA Tag	GGAGCTGTTATTGCTGCAGCTC	ACCAGCTCAATTAGTTGTAAGAGG
Mixl1	CCGCTTTCCCCATCTTCC	GACTTCCCAGCACCTCCACT
EphB2 LoxP site 1	AGGTAGGCACCACCATGATC	AGGCTGGCATGGGTTAGTTC
EphB2 LoxP site 1	GACCACTCCACCAGTAAAGAAAGG	CAAGCAGGATATGAGGGAGCAG
EphB2 LoxP site 1	GGCAGGTGGATCTCTGAGTTTG	CCACCCTGTGCTATCTATCAGTCA
Cftr	TCACAGCAATTTAAGTAGGGGC	GGGATGATACCGTCCATCTTGG

For NGS PCRs, primers contain Nextera adapter sequences at the 5’ end. The adapter sequences for the forward primer are TCGTCGGCAGCGTCAGATGTGTATAAGAGACAG. The adapter sequences for the reverse primer are GTCTCGTGGGCTCGGAGATGTGTATAAGAGACAG.

Supplementary Note S1. One-step digestion-ligation protocol using PEA1 to generate PE targeting constructs.

Oligos for guide and RT template insertion into plasmid need to be of the following form:

pegRNA guide:

```
5'-CACCGNNNNNNNNNNNNNNNNNNNN-3'
      3'-CNNNNNNNNNNNNNNNNNNNNCAAA-5'
```

pegRNA RT template

```
5'-GTGCNNNNNNNNNNNNNNNNNNNNNNNNNNNNNN-3'
      3'-NNNNNNNNNNNNNNNNNNNNNNNNNNNNNNAAAA-5'
```

Second-nick guide

```
5'-ACCGNNNNNNNNNNNNNNNNNNNNGT-3'
      3'-NNNNNNNNNNNNNNNNNNNNCAAAAT-5'
```

If the first N on the top strand for each guide is a G, it should be excluded.

- For the guides, the N's (typically 20 bases) in the two top strands comprise the guide sequence, which target the identical gRNA binding sequences followed by PAMs in the genomic DNA.
- The overhangs allow the oligos to bind the complementary overhanging DNA at the cut sites in the plasmid created by *Bbs*I digestion.
- The U6 promoter is more efficient if it starts transcription with a G, this is the reason for the extra G/C in the first pair of oligos, this doubles as the first base in the guide which is why it should be excluded if the guide starts with a G. The G is also present as part of the overhang in the second pair of oligos.
- The extra GT/CA in the second pair of oligos completes the gRNA scaffold.

1. Mix the following reagents in a PCR tube for each of the two inserts:

Reagent	Amount
MQ H_2O	6.5 µL
NEB T4 DNA Ligase Buffer with 10 mM ATP (10x)	1 µL
top oligo (100 µM)	1 µL
bottom oligo (100 µM)	1 µL
NEB T4 PNK (10 U/µL)	0.5 µL
**Total**	**10 µL**

2. Place each mixtures in thermocycler with the following parameters:

**1**	37 °C	30 min
**2**	95 °C	5 min
**3**	Ramp to 25 °C @ 0.1 °C/s	∞

3. Dilute the 3 sets of phospho-annealed oligos 1:250 with MQ H_2O in a 1.5 mL tube.

Reagent	Amount
MQ H_2O	249 µL
phospho-annealed oligo	1 µL
**Total**	**250 µL**

4. Mix the following reagents in a PCR tube:

Reagent	Amount
MQ H_2O	10 µL
PEA1 empty plasmid (100 ng/µL)	1 µL
phospho-annealed oligo pair 1 (1:250)	1 µL
phospho-annealed oligo pair 2 (1:250)	1 µL
phospho-annealed oligo pair 3 (1:250)	1 µL
NEBuffer 2.1 (10x)	2 µL
DTT (10 mM)	1 µL
ATP (10 mM)	1 µL
NEB *Bbs*I (5 U/µL)	1 µL
T4 DNA Ligase (400 U/µL)	1 µL
**Total**	**20 µL**

Note: We showed that removing NEBuffer 2.1, DTT, ATP components and replacing it with 2 ul of 10x T4 Ligase Buffer (add extra MQ to reach total volume of 20 ul) could lead to successful reaction.

5. Place in thermocycler with the following parameters:

**1**	37 °C	5 min
**2**	16 °C	5 min
**3**	Go to step 1	5 times

6. Transform to competent cells.
   Recommended: Incubating the reaction overnight at 4 C before transformation could lead to higher number of colonies.

7. Check digest using Bbs1. Plasmids with complete integrations remain circular.
8. Sequence verify using primers GGTTTCGCCACCTCTGACTTG and CACTCCCACTGTCCTTTCCTAATA.

Supplementary Note S2. Generation of pegRNA protocol.

Kit: NEB HiScribe™ T7 Quick High Yield RNA Synthesis.

T7 guide primer for PCR of gRNA oligo needs to be of the following form:

5’-TTAATACGACTCACTATAGNNNNNNNNNNNNNNNNNNN-3’

Where the N’s are identical to your specific guide.

Reverse primer for PCR of gRNA oligo needs to be:

5’- AAAANNNNNNNNNNNNNNNNNNNNNNNNNN-3’

Where the N’s are the RT template sequence.
This oligos are the same oligos used for RT template bottom oligos in the digestion-ligation protocol.

1. Generate the relevant PEA1 targeting plasmid (miniprep)
2. Mix the following master mix reagents in a 1.5 mL tube and aliquot into 6 PCR tubes:

Reagent	Amount	6x MM
MQ H_2O	12.3 µL	73.8 µL
NEB Phusion HF Reaction Buffer (5x)	4 µL	24 µL
T7 guide primer (10 µM)	1 µL	6 µL
Reverse primer (10 µM)	1 µL	6 µL
NTP Mix (10 mM)	0.5 µL	3 µL
NEB Phusion HF DNA Polymerase (2 U/µL)	0.2 µL	1.2 µL
1 µL PEA1 targeting plasmid (~1-3 ng/µL)	1	6
**Total**	**20 µL**	**120 µL**

3. Place the tubes in a thermocycler with the following parameters:

**1**	98 °C	3 min
**2**	98 °C	15 s
**3**	60 °C	20 s
**4**	72 °C	15 s
**5**	Go to step 2	32 times
**6**	72 °C	5 min
**7**	4 °C	∞

4. Make a 1% agarose gel and run 5 µL of the PCR products

Testing the plasmid has the correct insert.

Band should be present at ~100 bp.

5. Combine all PCR reactions and perform **Qiagen PCR Purification** in a single column.
6. Use NanoDrop to measure concentration of DNA.

Confirms the DNA is still present.

7. Perfrom IVT by mixing the following reagents in a PCR tube:

Reagent	Amount
Nuclease-free MQ H_2O	up to 40 µL
NEB NTP Buffer Mix (20 mM)	20 µL
Purified PCR product	~1000 ng
NEB T7 RNA Polymerase Mix	4 µL
**Total**	**~40 µL**

Note: half reaction (total 20 ul) is also possible.

8. Incubate O/N @ 37 °C in thermocycler.

Note: 3 hours is also possible.

9. Transfer 2 µL to PCR tube for testing later.
10. Mix the following reagents in a PCR tube:

Reagent	Amount
Nuclease-free MQ H_2O	60 µL
IVT gRNA product	40 µL
NEB DNase I (RNase-free) (2 U/µL)	4 µL
**Total**	**104 µL**

Degrades DNA.

11. Incubate 15 min @ 37 °C.
12. Transfer 2 µL to PCR tube for testing later.
13. Perform **Qiagen RNEasy Mini Kit RNA Cleanup**, eluting in 30 µL.
14. Check RNA on gel (RNAse free technique should be applied).

Supplementary Note S3. Generation of nuclease prime editor mRNA protocol.

- Linearize plasmid PE2-Nuc using Pme1
    - MQ = X ul
    - Cut smart buffer = 6 ul
    - Plasmid = Y ul (10 ug)
    - Pme1 = 3 ul
    - Total 60 ul
    - Incubate 37 C for 2 hours
- Purify the linearized plasmid using Zymo DNA clean and concentrator 5
    - Add 200 ul binding buffer
    - Spin
    - Add 200 ul wash buffer spin
    - Repeat wash
    - Add 12 ul RNAse-free water
    - Spin
- Setup IVT using Mmessage ultra kit
    - T7 Arca = 10 ul
    - Buffer = 2 ul
    - Linearized plasmid = X ul (1.5-2 ug)
    - T7 enzyme = 2 ul
    - RNAse-free water = Y ul (total 20 ul)
    - Incubate 37 C for 3 hours
    - Add 1 ul of DNAse, incubate 30 min 37 C
    - Add 36 ul water + 20 ul EPAP + 10 ul MnCl2 + 10 ul ATP (all included in the kit)
    - Take 2.5 ul for gel checking
    - Add 4 ul of EPAP enzyme, incubate 37 C for 20 min
    - Keep the reaction on ice
    - Take 2.5 ul for gel checking
    - Proceed to RNA clean up using RNAeasy kit (elute in 35 ul of water).
- Zygote microinjection mix
    - MQ = X ul
    - 10x injection buffer = 1.5 ul
    - Nuclease prime editor mRNA = Y ul (final 150 ng/ul)
    - pegRNA = Z ul (final 75 ng/ul)
    - Total = 15 ul

10X injection buffer

Reagent	Amount
pH 8.0 EDTA (0.5 M)	10 µL
pH 7.5 Tris (1 M)	500 µL
Nuclease-free MQ H_2O	4.49 mL
**Total**	**5 mL**

(Filtered into aliquots in 1.5 mL tubes and stored @ - 20 °C.)

Supplementary Note S4. Data analysis using Rgenome PE-Analyzer.

%Correct PE
Correct PE can be directly gathered from the generated analysis

Prime editing
64 (1.4%)

%Unmodified (WT)
WT frequency can be gathered directly by clicking the WT column. Ensure you don't filter any sequence here.

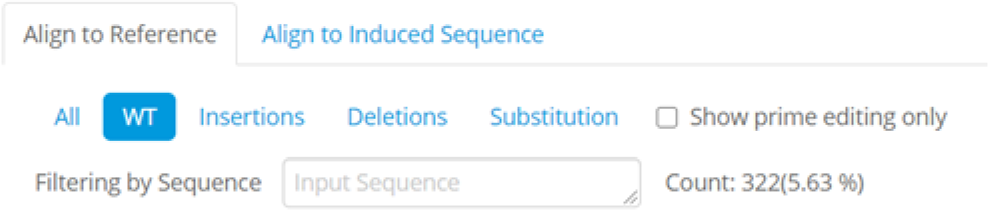

%Unintended edits = 100 - %Correct PE - %WT

%Any intended edits: any alleles containing prime edited sequences (modified and/or unmodified)
To get total PE, filter sequences starting from PBS to 2nt of the edit.

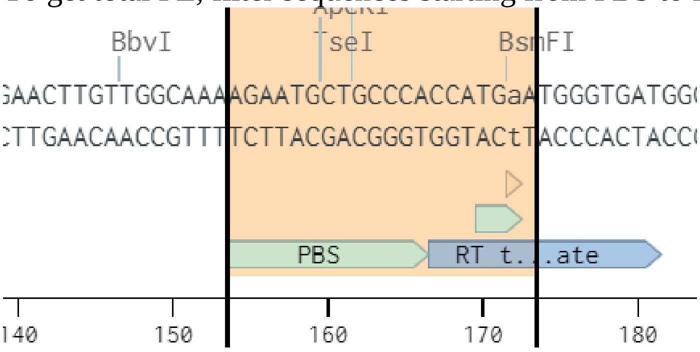

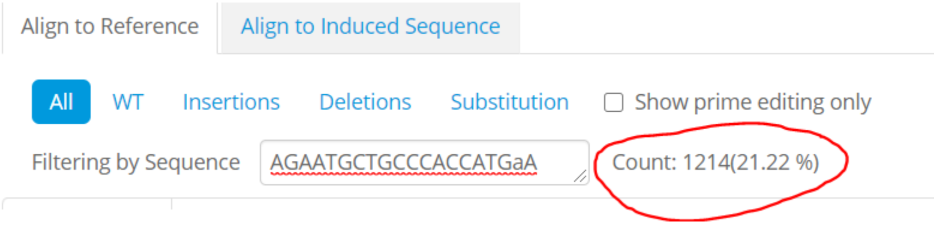

The sequence list to filter to get total PE:
HEK3:
+1 A ins: CAGACTGAGCACGaT
+1 CTT ins: CAGACTGAGCACGct
+1 T del: CAGACTGAGCACGGA
+1-3 TGA del: CAGACTGAGCACGTGG
+1 T to G PM: CAGACTGAGCACGgG
+2 G to C PM: CAGACTGAGCACGTcA

RNF2:
+1 T ins: GaGTAATGACTAAGATG
+1 GTA ins: acGTAATGACTAAGATG
+4 A del: CCCAGGTAATGACTAAGATG
+3-5 GAG del: ACAGGTAATGACTAAGATG
+1 C to G PM: AcGTAATGACTAAGATG

+2 T to A PM: CtGGTAATGACTAAGATG

RUNX1:
+1 C ins: ATTTTCAGGAGGAAGcC
+1 ATG ins: ATTTTCAGGAGGAAGCat
+2 G del: ATTTTCAGGAGGAAGCAT
+2-4 GAT del: ATTTTCAGGAGGAAGCGG
+1 C to G PM : ATTTTCAGGAGGAAGgG
+3 A to T PM : ATTTTCAGGAGGAAGCGtT

VEGFA:
+4 C ins:TCTGCAGGCCAGATGAGcG
+2 ACA ins: TCTGCAGGCCAGATGac
+3 A del: TCTGCAGGCCAGATGGG
+2-4 GAG del: TCTGCAGGCCAGATGG
+1 T to G PM: TCTGCAGGCCAGAgG
+2 G to A PM: TCTGCAGGCCAGATaA

Mouse ES cells
Mixl1 +1 CTT ins: agACCCAGACATCCAC
Mixl1 +1-3 ACA del : CAACCCAGACATCCAC
Mixl1 +1 A to G : GcACCCAGACATCCAC

Tyr +1 TGT ins : AGAATGCTGCCCAtg
Tyr +6 G to A : AGAATGCTGCCCACCATGaA

Chd2 +1 CTC ins : agGTTCTGGGAGCTA
Chd2 +5 G to C : CgCACCGTTCTGGGAGCTA

Col12a1 +1 GTG ins : TTCCATGGTTCCAgt
Col12a1 +2 A to C : TTCCATGGTTCCACcA

%Any loxP: any alleles containing loxP sequences (modified and/or unmodified)
LoxP site 1 ATAACTTCGTATAATGTATGCTATACGAAGTTATcaattg
LoxP site 2 ATAACTTCGTATAATGTATGCTATACGAAGTTATgatatc
LoxP site 3 ATAACTTCGTATAATGTATGCTATACGAAGTTATgatatc

%Partial template duplications (PTDs)
To get the frequency of PTDs, filter the same sequences above into the "insertions" column.

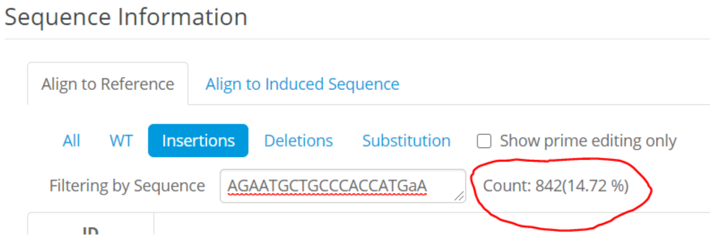

If the edit is substitution or deletion, collect the frequency of PTD straight away from the count.

If the edit is insertion, the PTD = the count – the correct PE.

%Indels = %unintended edits - %PTDs
